# Preoperative vestibular assessment protocol of cochlear implant surgery: an analytical descriptive study^☆^

**DOI:** 10.1016/j.bjorl.2016.06.014

**Published:** 2016-07-31

**Authors:** Roseli Saraiva Moreira Bittar, Eduardo Setsuo Sato, Douglas Jósimo Silva Ribeiro, Robinson Koji Tsuji

**Affiliations:** Universidade de São Paulo (USP), Escola de Medicina, Departamento de Otorrinolaringologia, São Paulo, SP, Brazil

**Keywords:** Cochlear implant, Vestibular function, Preoperative diagnosis, Implante coclear, Função vestibular, Diagnóstico pré-operatório

## Abstract

**Introduction:**

Cochlear implants are undeniably an effective method for the recovery of hearing function in patients with hearing loss.

**Objective:**

To describe the preoperative vestibular assessment protocol in subjects who will be submitted to cochlear implants.

**Methods:**

Our institutional protocol provides the vestibular diagnosis through six simple tests: Romberg and Fukuda tests, assessment for spontaneous nystagmus, Head Impulse Test, evaluation for Head Shaking Nystagmus and caloric test.

**Results:**

21 patients were evaluated with a mean age of 42.75 ± 14.38 years. Only 28% of the sample had all normal test results. The presence of asymmetric vestibular information was documented through the caloric test in 32% of the sample and spontaneous nystagmus was an important clue for the diagnosis. Bilateral vestibular areflexia was present in four subjects, unilateral arreflexia in three and bilateral hyporeflexia in two. The Head Impulse Test was a significant indicator for the diagnosis of areflexia in the tested ear (*p* = 0.0001). The sensitized Romberg test using a foam pad was able to diagnose severe vestibular function impairment (*p* = 0.003).

**Conclusion:**

The six clinical tests were able to identify the presence or absence of vestibular function and function asymmetry between the ears of the same individual.

## Resumo

**Introdução:**

Os implantes cocleares (IC) são indiscutivelmente um método eficaz de recuperação da função auditiva de pacientes surdos.

**Objetivo:**

Descrever o protocolo de avaliação vestibular pré-operatória em sujeitos que serão submetidos ao IC.

**Método:**

Nosso protocolo institucional prevê o diagnóstico vestibular por meio de seis testes simples: testes de Romberg e Fukuda, nistagmo espontâneo, *Head Impulse Test*, *Head Shaking Nistagmus*, prova calórica.

**Resultados:**

Foram avaliados 21 pacientes com idade média de 42,75  ±  14,38 anos. Apenas 28% da amostra apresentou todos os testes normais. A presença de informação vestibular assimétrica foi documentada pela prova calórica em 32% da amostra e o nistagmo espontâneo mostrou-se pista importante para seu diagnóstico. A arreflexia vestibular bilateral foi diagnosticada em quatro sujeitos; arreflexia unilateral em três e hiporreflexia bilateral em dois. O *Head Impulse Test* mostrou-se indicador significante (*p* = 0,0001) para diagnosticar arreflexia da orelha testada. O teste de Romberg sensibilizado em almofada foi capaz de diagnosticar os comprometimentos severos da função vestibular (*p* = 0,003).

**Conclusão:**

Os seis testes clínicos foram capazes de identificar a presença ou não de função vestibular e assimetria da função entre as orelhas de um mesmo indivíduo.

## Introduction

Cochlear implants (CI) are highly effective devices for recovery of hearing function in individuals with hearing loss and have facilitated integration into social life. The success of post-implant rehabilitation has raised new challenges in both the selection and the planning of the hearing prognosis of subjects undergoing surgery. Although the cochlear system is distinct from the vestibular system, both have identical neural transmission. The benefits of the electrical stimulation of the CI go beyond the auditory pathways and also benefit the vestibular system and postural control.^1^ Nonetheless, the CI is not without risk to the semicircular canal and otolith function and may impair or suppress vestibular function, especially if there is pre-existing pathology.

The prevalence of postoperative dizziness varies widely in the literature and is around 20% in our cases. It usually resolves in approximately 30 days. Some of these patients develop bilateral vestibular areflexia (BVA), which severely reduces patient quality of life.^2^ Knowledge of vestibular system function before and after CI surgery is important for the satisfactory management of each case. Therefore, we have added vestibular assessment to our outpatient routine prior to CI surgery. Our main goal is to document the existence of vestibular function and possible asymmetries between the ears before surgery. This information can help in the selection of which ear to implant and can assist in the management of any postoperative vestibular symptoms.

In adults, preoperative vestibular assessment was designed to be brief and easy to perform, using resources available at any otorhinolaryngology outpatient clinic. The tests used are able to identify both vestibular asymmetry from unilateral lesions as well as bilateral involvement. The protocol was designed to be accessible to services that perform CI surgery but do not always have a neurotological department and research equipment.

It is not our intention to speak at length about each vestibular test used, but to provide the reader a quick and convenient method to identify vestibular impairment. Knowledge of vestibular function can indicate the adequate management and prevent undesirable side effects.^3^, ^4^

Our goal is to describe the preoperative vestibular assessment of adult patients who are cochlear implant candidates in our institution, demonstrate its effectiveness and discuss its importance in the postoperative outcome.

## Methods

This is a descriptive and analytical cross-sectional study that followed the ethical standards approved by the CAEPesq number 0983.07. All participants are from the Institution Otorhinolaryngology Clinic.

Our sample comprises 21 subjects, 10 men and 11 women, mean age of 46 ± 14.74 years who agreed to participate in the study. All adults previously selected for CI surgery, admitted between May 2013 and November 2014 were included in the sample. All patients were capable of understanding and performing the necessary examinations for the preoperative vestibular diagnosis. The assessment includes six tests that identify asymmetries, the affected sides or the complete absence of vestibular function. The study sample including their ages and etiologies are shown in Table 1.

**Table 1:** List of assessed patients regarding gender, age and etiology of hearing loss.

	Gender	Age	Etiology of hearing loss
SLNSJ	M	20	Meningitis
ADPDJ	M	21	Meningitis
FDCDSCF	M	27	Traumatic brain injury (TBI)
KPG	F	28	Unknown
ROR	F	29	Unknown
LTF	M	32	Meningitis and TBI
GFR	M	35	TBI
AMPDS	M	38	Meningitis
LJAC	F	42	Unknown
LALM	F	45	Unknown
DAA	M	45	Meningitis
GBP	F	45	Measles
SM	F	45	Chronic otitis media and TBI
RDCCDS	F	45	Toxoplasmosis
MSC	F	46	Genetic hearing loss
EBDS	F	48	Meningitis
ACDS	F	49	Unknown
MCB	F	51	Unknown
JONDC	M	63	Otosclerosis
FFT	M	66	Chronic otitis media
PLDS	M	77	Otosclerosis

### VOR and vestibular asymmetry assessment^5^

#### Spontaneous nystagmus^6^

Spontaneous nystagmus (SN) is ocular movement observed with the patient in the sitting position with the eyes fixed straight ahead. The presence of SN indicates vestibular asymmetry and is the results of asymmetry in oculomotor tone that originated from the vestibular system. It consists in a slow gaze deviation, followed by a quick movement of the eyeball to the center position (corrective saccades). When it has a peripheral origin, spontaneous nystagmus decreases with eye fixation and intensifies when the eye is diverted 30° toward the fast component (Alexander's Law).^6^ The direction of the nystagmus is the direction of the corrective saccade which is easier to visualize. But it is the slow gaze deviation that determines the side with hypofunctioning labyrinth.

### Head Impulse Test (HIT)^7^

The Head Impulse Test is a simple clinical maneuver to identify an impaired vestibulo-ocular reflex in the tested ear by observing the ocular response that occurs after a quick head movement. While facing the examiner, the patient focuses on a target on the examiner's face. The examiner holds the patient's face in his/her hands and suddenly turns his/her head, and observes the ocular response. A normal individual will keep his/her eyes on the target, but when the vestibulo-ocular reflex (VOR) is impaired, the eyes will follow the movement of the head and then perform a saccade in the opposite direction to the corrective head movement to re-fixate on the visual target.

### Head Shaking Nystagmus^8^, ^9^

The Head Shaking Nystagmus (HSN) examination investigates asymmetry of muscle tone at high frequencies of head rotation. The patient sits face-to-face with the examiner and looks at a target on the examiner's face. Then the patient performs 20 lateral head turns at high speed. At the end of the test, the patient interrupts the rotations, stabilizes the head and remains facing the front direction. The presence of nystagmus indicates asymmetry of vestibular information. In peripheral labyrinthine disorders, the slow gaze deviation indicates a hypofunctioning labyrinth.

### Caloric testing^5^

Caloric testing is performed in the supine position with the head flexed 30°. Water at 30° and 44° is the stimulus used for the test. Irrigations are performed in both ears with a five-minute interval between them. The resulting nystagmus is recorded using nystagmography equipment. Normal angular velocities of nystagmus are between 7° and 52°. When the angular velocity of the movements exceeds these limits, it is called hyperreflexia and when it is below the minimum, hyporeflexia. The equipment used in this study was the vector-electronystagmography SCE Contronic^®^.

Labyrinthine predominance (LP) designates asymmetry between the vestibular responses. A value of 18% was chosen for LP. The presence of LP means asymmetry of vestibular information of peripheral or central origin and always indicates the side with better function.

### Postural test assessment

Alterations of the Romberg's and Fukuda's tests are not pathognomonic of vestibular pathology, although they help with the diagnosis. The tests can be influenced by proprioceptive or neurological lesions and should be used in conjunction with other vestibular tests.

### Romberg's test^10^

The patient is instructed to stand with heels together and toes apart at approximately 30°. The arms can rest along the body or be extended forward. The test is considered positive for vestibular asymmetry if the patient shifts or falls. The side of the fall will be the side with labyrinth hypofunction.

**Figure 1:**
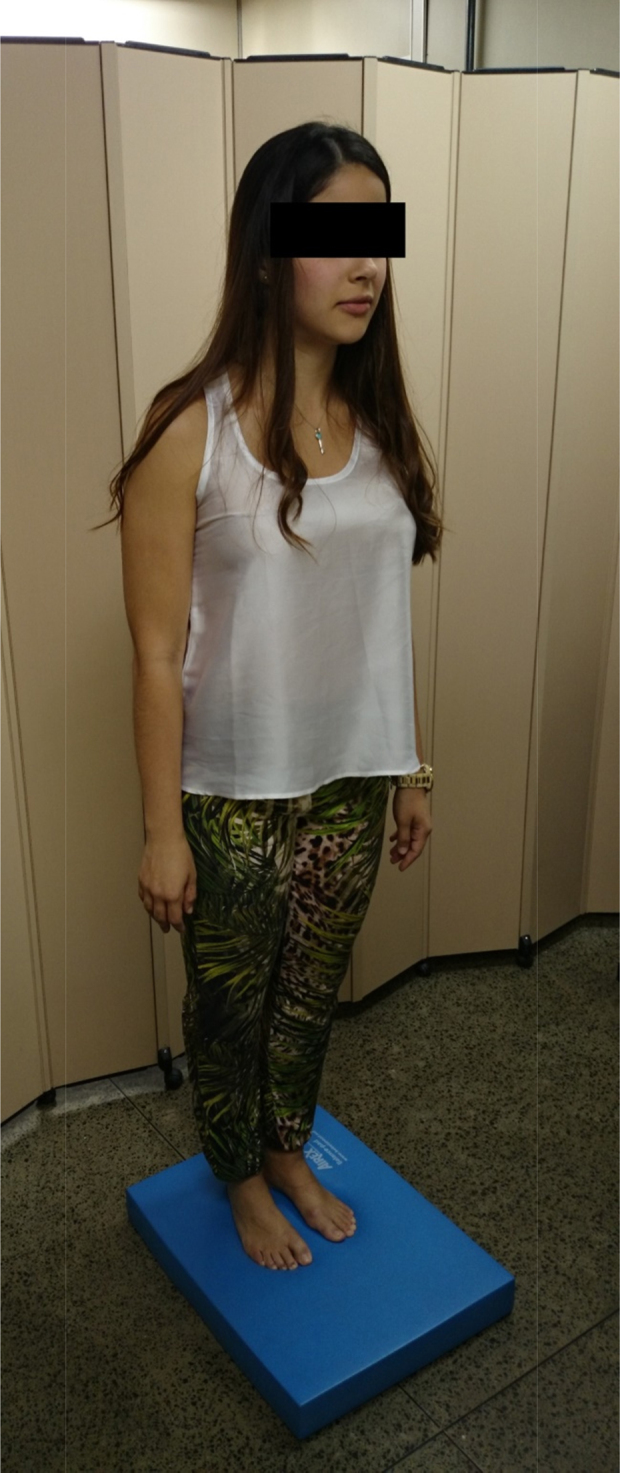
Test using a foam pad.

The sensitivity of the test can be increased if it is performed on a foam pad, first with the eyes open and then with the eyes closed. The test is positive when a subject can stabilize the posture with the eyes open, but not with them closed. The test on the pad simulates condition 5 of the dynamic posturography and the subject's fall indicates absence of vestibular information^11^ (Fig. 1).

### Fukuda's test^12^

Fukuda's test is used to identify tone asymmetries in the distal lower-limb muscles. With arms outstretched, the patient is asked to march 60 steps with the eyes closed and without moving. The subject's angles of deviation and shifting are evaluated. At the end of the test, an angular deviation of up to 30° and the linear displacement of up to 50 cm are considered normal. The posterior displacement in relation to the starting point is not common. Rotations higher than 45° are considered abnormal. Asymmetrical lesions of the vestibular system result in body rotation in the direction of the slow nystagmus component – the hypofunctioning labyrinth.

Test results were quantified as percentages and associated using Fisher's exact test and *p*-values ≤0.05 for a 95% confidence interval were considered statistically significant.

## Results

The mean time taken to perform all the tests was approximately 1 h. Of the 21 patients, only 6 (28%) had normal results in all tests. Therefore, 72% of the sample showed some type of alteration during vestibular assessment.

Regarding information asymmetry, we identified 10 (32%) subjects with the presence of post-caloric test asymmetry. Of these, 2 (20%) had spontaneous nystagmus – one case was associated with the presence of labyrinthine predominance, while the other was not. Three subjects with post-caloric asymmetry showed complete lack of response in one ear. Among these last three, two fell and showed great instability at the Romberg test using a foam pad and with eyes closed (*p* = 0.008) (Table 2).

**Table 2:** Significance of the incidence of falls at the Romberg test using a foam pad in relation to the absence of vestibular function in one ear in subjects with asymmetric post-caloric function (Fisher's exact test).

	Falls	No falls	Total
Asymmetric without areflexia	0	7	7
Unilateral vestibular areflexia	3	0	3
Total	3	7	10

Fisher's test, *p* = 0.008.

Still considering the caloric test, 1 subject (4.5%) had bilateral hyporeflexia and 4 (18%) bilateral areflexia. All patients (100%) fell during the Romberg test using a foam pad with closed eyes. Fisher's exact test shows an association between the fall at Romberg test using a foam pad with closed eyes and areflexia or severe vestibular function impairment (*p* = 0.003) (Table 3).

**Table 3:** Significance of the incidence of falls at the Romberg test using a foam pad in relation to the presence or absence of residual vestibular function (Fisher's exact test).

	Falls	No falls	Total
Present vestibular function	3	13	16
Absent vestibular function	5	0	5
Total	8	13	21

Fisher's test, *p* = 0.003.

When individually analyzing vestibular function in each ear, the four subjects who had bilateral areflexia (8 ears) and three with unilateral arreflexia (three ears) at the caloric test, had a positive HIT result in 6 (54%) ears. HIT was considered highly significant to identify areflexic ears (*p* = 0.0001) (Table 4).

**Table 4:** Significance of a positive Head Impulse Test (HIT) and absence of function (areflexia) of the assessed ears (Fisher's exact test).

	Positive HIT	Negative HIT	Total
Areflexic ears	8	3	11
Functional ears	0	31	31
Total	8	34	42

Fisher's test, *p* = 0.0001.

The HSN test did not show any abnormal results in the assessed patients.

## Discussion

Balance is a vital condition for the preservation of our species. To adequately perceive the environment around us and to react to postural challenges are necessary for body safety. The structural and functional integrity of the vestibular system is necessary for maintenance of the entire complex postural system, adaptation to the environment, and the fight-or-flight response. The absence of vestibular function is accompanied by poor prognosis and severe limitations in the activities of daily life, such as ambulating in low-light environments or on uneven ground, swimming, driving fast, etc. A frequent complaint is oscillopsia during head movements, especially in the dark.^13^ The benefits of the CI are proven and its positive impact on deaf patients’ auditory perception and quality are no longer debated. However, the surgical implant procedure requires the opening of the labyrinth and there are risks when the vestibular function is present. Prior knowledge of the vestibular condition increases the diagnostic index and helps the management of possible postoperative vestibular complications. There are two questions that should be raised when we consider the vestibular function of a patient who will be submitted to CI: (1) Is vestibular function present? (2) Is the function symmetric? To answer these questions we developed a simple preoperative assessment that does not require sophisticated equipment.

The importance of the preoperative assessment can be understood when we observe our results, which identified 72% of the sample with some type of vestibular alteration. In case of normal function, when one ear is implanted, it is possible to reverse the dizziness symptom, even if there is complete loss of vestibular function on the operated side. The adequate use of neuroplasticity mechanisms can restore body balance through vestibular rehabilitation techniques. The cases that offer greater risk of permanent lesion are those with vestibular function asymmetry between the ears. Adequate vestibular assessment prevents bilateral vestibular areflexia, as it provides data on the presence or absence of vestibular function and allows choosing the ear that offers lower surgical risk.

We identified 10 (32%) subjects with post-caloric test asymmetry. In these cases, spontaneous nystagmus was present in 20%, indicating the presence of vestibular function asymmetry at the caloric test.

Another important test to detect vestibular tone asymmetry is the HSN test. However, there is difficulty in observing the head-shaking nystagmus, especially because patients fix their gaze on the examiner's face at the end of the procedure. It is a known fact that ocular fixation inhibits nystagmus of peripheral origin. None of our patients had a positive HSN test, but whether the nystagmus inhibition caused by ocular fixation would not have prevented the visualization of the final nystagmus is yet to be clarified. The use of Frenzel glasses decreases the bias by preventing ocular fixation.

The patients evaluated in this sample were not submitted to Fukuda test, subsequently added to the diagnostic routine. The test is useful to assess deviations secondary to tone asymmetry and it is routinely used to evaluate vestibular compensation during treatment. We decided to include it subsequently to observe the association between vestibular tone asymmetry diagnosed by spontaneous nystagmus and post-caloric asymmetries – important for choosing the ear to be implanted. In such cases, both the spontaneous nystagmus and the Fukuda test contribute important information when searching for unilateral lesions.

The worst post-operative situation is bilateral vestibular areflexia (BVA), as vestibular rehabilitation is limited, only improving balance by 50% in these cases.^2^ BVA can be prevented when there is prior knowledge of the vestibular function of the ear to be implanted. Classically, BVA can be easily diagnosed by fall in condition 5 of the computerized dynamic posturography, which subjects the individual to oscillation of foot support with closed eyes. Condition 5 can be perfectly mimicked by the Romberg test using a foam pad. In this situation, without the vision and with conflicting proprioceptive information, the only determinant of posture is the vestibular function and, in its absence, the fall occurs.^14^, ^15^

Patients with post-caloric asymmetry due to absence of vestibular responses on one side also showed a low performance on the Romberg test using a foam pad: 66% fell and 33% showed severe instability. Statistical analysis of the sample shows that the Romberg test using a foam pad was sensitive to diagnose severe vestibular function impairment (*p* = 0.003). Our data are consistent with the literature that attributes 79% of sensitivity and 80% of specificity to the test when detecting unilateral or bilateral arreflexia.^16^

In those patients who had no vestibular response to the caloric test, the HIT was valuable (*p* = 0.0001). A positive test result is highly suggestive of failure of the vestibulo-ocular reflex, with the additional advantage of identifying the affected side.^7^ The diagnosis of vestibular areflexia in the ear to be implanted makes the surgeon more confident, as there is no function to be compromised. In bilateral cases, there is no risk of postoperative dizziness.

Finally, it is important to remember that the knowledge of the vestibular function prior to the surgery allows the physician to assess what occurred during surgery.^17^ It will be possible to say whether there was a lesion in the operated side or if a pre-existing situation was aggravated. A simple, six-step evaluation before surgery offers the possibility of identifying lesions and being prepared to manage any vestibular impairments.

## Conclusion

We concluded that 72% of the patients presented unilateral or bilateral vestibular lesions. The HIT associated with the Romberg test with foam pad was highly sensitive to diagnose severe impairment of the vestibular function, confirmed by the caloric test.

## Conflicts of interest

The authors declare no conflicts of interest.
